# A Community Multi-Omics Approach towards the Assessment of Surface Water Quality in an Urban River System

**DOI:** 10.3390/ijerph14030303

**Published:** 2017-03-14

**Authors:** David J. Beale, Avinash V. Karpe, Warish Ahmed, Stephen Cook, Paul D. Morrison, Christopher Staley, Michael J. Sadowsky, Enzo A. Palombo

**Affiliations:** 1 Land and Water, Commonwealth Scientific and Industrial Research Organization, P.O. Box 2583, Dutton Park, Queensland 4001, Australia; akarpe@swin.edu.au (A.V.K.); Warish.Ahmed@csiro.au (W.A.); 2 Department of Chemistry and Biotechnology, Faculty of Science, Engineering and Technology, Swinburne University of Technology, P.O. Box 218, Hawthorn, Victoria 3122, Australia; epalombo@swin.edu.au; 3 Land and Water, Commonwealth Scientific and Industrial Research Organization, Private Bag 10 Clayton South, Victoria 3169, Australia; Stephen.Cook@csiro.au; 4 Australian Centre for Research on Separation Science, School of Applied Sciences, RMIT University, P.O. Box 2547, Melbourne, Victoria 3000, Australia; Paul.Morrison@rmit.edu.au; 5 Biotechnology Institute, University of Minnesota, St. Paul, MI 55108, USA; cmstaley@umn.edu (C.S.); sadowsky@umn.edu (M.J.S.)

**Keywords:** contaminated system, urban river system, metagenomics, metabolomics, trace metals, chemometrics

## Abstract

A multi-omics approach was applied to an urban river system (the Brisbane River (BR), Queensland, Australia) in order to investigate surface water quality and characterize the bacterial population with respect to water contaminants. To do this, bacterial metagenomic amplicon-sequencing using Illumina next-generation sequencing (NGS) of the V5–V6 hypervariable regions of the 16S rRNA gene and untargeted community metabolomics using gas chromatography coupled with mass spectrometry (GC-MS) were utilized. The multi-omics data, in combination with fecal indicator bacteria (FIB) counts, trace metal concentrations (by inductively coupled plasma mass spectrometry (ICP-MS)) and in-situ water quality measurements collected from various locations along the BR were then used to assess the health of the river ecosystem. Sites sampled represented the transition from less affected (upstream) to polluted (downstream) environments along the BR. Chemometric analysis of the combined datasets indicated a clear separation between the sampled environments. *Burkholderiales* and *Cyanobacteria* were common key factors for differentiation of pristine waters. Increased sugar alcohol and short-chain fatty acid production was observed by *Actinomycetales* and *Rhodospirillaceae* that are known to form biofilms in urban polluted and brackish waters. Results from this study indicate that a multi-omics approach enables a deep understanding of the health of an aquatic ecosystem, providing insight into the bacterial diversity present and the metabolic output of the population when exposed to environmental contaminants.

## 1. Introduction

Understanding the complex interaction between microbial communities and environmental changes, natural or anthropogenic, is a major research challenge [1]. Free-living microorganisms, such as planktonic bacteria in a water column or the diverse microbial community found in soils, would be predicted to display large inter-individual metabolic variability as a result of their individual state, interaction with their surrounding environment and exposure to pollution sources. However, through a systems biology approach, harnessing the depth of knowledge provided by each microorganism can be aggregated and investigated in greater detail, and with increased throughput [2,3]. For example, microbial communities are known to colonize most environments, including contaminated sites or harsh climatic environments [4,5,6]. These communities have been demonstrated to metabolize recalcitrant contaminants (via bioremediation) and have been observed to thrive under extreme conditions [7]. The recent development of omics-based techniques has enabled environmental microbiologists to recognize and characterize such microbial communities as an imperative ecological parameter in monitoring polluted and extreme environments, either by detecting community shifts in response to contaminant(s) or their resilience towards climatic or physico-chemical disturbances [7]. In this context, the application of metagenomics and meta-transcriptomics has been vital in detailing the microbial community present and its potential activity, and techniques such as meta-proteomics and metabolomics have provided insight into the specific proteins and metabolites that are expressed [2].

Desai et al. [7], amongst others, proposed that the simultaneous analyses of multiple omics-based approaches would lead to a system-wide assessment of site-specific microorganisms and their underlying physico-chemical disturbances; they focused on characterizing the protein synthesis and carbon and carbohydrate metabolism of the sampled population at xenobiotic/anthropogen contaminated sites. To an extent, many researchers have already started employing multi omics-based techniques to their research. Bullock et al. [8] investigated the microbial activity in relation to organic matter degradation and turnover at various locations in the Mid-Atlantic Bight. Date et al. [9] used metagenomics and ^13^C labelled metabolomics to monitor the metabolic dynamics of fecal microbiota. Hook et al. [10] investigated contaminated sediments using transcriptomics and metabolomics in order to better understand the modes of toxic action within contaminated ecosystems. Specifically, the function of transcripts with altered abundance of *Melita plumulosa* (an epibenthic amphipod) was investigated following whole-sediment exposure to a series of common environmental contaminants. Such contaminants included pore-water ammonia, bifenthrin and fipronil (pesticides), diesel and crude oil (petroleum products), and metals (Cu, Ni, and Zn). Subsequent data integration and hierarchical cluster analysis demonstrated grouped transcriptome and metabolome expression profiles that correlated with each specific contaminant class. Many of the transcriptional changes observed were consistent with patterns previously described in other crustaceans [11]. Likewise, Hultman et al. [5] undertook a similar study investigating the microbial metabolism of permafrost. They used several omics approaches, combined with post-data analysis, to determine the phylogenetic composition of microbial communities of intact permafrost, the seasonally thawed active layer and thermokarst bog (surfaces of marshy hollows). The multi-omics strategy revealed good correlation of process rates for methanogenesis (the dominant process), in addition to providing insights into novel survival strategies for potentially active microbes in permafrost [5].

The inclusion of metabolomics in (meta)transciptomics and metagenomics investigations has enabled researchers to assess biochemical profile variations of entire microbial communities living in contaminated sites [6,12]. Metabolomics is a well-established scientific field that focuses on the study of low molecular weight metabolites (typically <1000 Da) within a cell, tissue or bio-fluid [13,14,15]. Furthermore, the application of environmental metabolomics is an expanding field within the metabolomics platform. Environmental metabolomics assesses and characterizes the interactions of living organisms within their environment [4] and is traditionally used as a tool to investigate environmental factors, either physical or chemical, and their impact to a specific organism. For example, Gómez-Canela et al. [16] used targeted environmental metabolomics to investigate *Gammarus pulex* (a freshwater amphipod crustacean) following controlled exposures to selected pharmaceuticals in water. Similarly, Cao et al. [17] studied the bioaccumulation and metabolomics responses in *Crassostrea hongkongensis* (an oyster) impacted by different levels of metal pollution; and Ji et al. [18] studied the impact of metal pollution on *Crangon affinis* (a shrimp). In addition, community metabolomics extends the application of environmental metabolomics even further through the investigation of all metabolites expressed from an entire microbial community, thus enabling a meta-metabolomics approach [6].

The advancement of omics-based techniques and their integration (coined multi-omics) have contributed towards the fields of environmental and molecular biology, thereby pushing the boundaries of our understanding of microbial physiology [19]. To date, such studies have focused on specific pollution events (e.g., the Deepwater Horizon oil spill [20]), the assessment of biotechnology/bioremdiation (e.g., bioremdiation of steriods in the enviornment [21]) or used to characterize well-controlled engineered systems (e.g., anerobic bioreactors [22,23]). To the best of our knowledge, such an approach has not been used to characterize a system as part of a water quality monitoring survey. The application of metagenomics or metabolomics in isolation has been applied with some success [24]. For example, metagenomics has been applied to assess drinking water microbial populations after various treatment methods [25] and assess river microbiomes across various land use types [26]. Beale et al. [27] used metabolomics with physico-chemical data to assess water pipeline infrastructure and water pipe biofilms, characterizing biofilms based on pipe material and the excreted metabolites that pass from the biofilm into the water stream. A similar study was used to investigate impacts of exposure to chemicals of emerging concern relative to other stressors in fathead minnows, which was used as a model species [28].

The current study herein merges bacterial metagenomics and community metabolomics with additional phyico-chemico data, thus using a multi-omics based approach to investigate an urban river system. It is anticipated that such an approach would provide an additional layer of information on top of traditional water quality monitoring parameters that will ultimately result in a deeper understanding of the the diverse microbial population present, enabling researchers to characterize environmental systems, not based on inferred water quality data but as an interconnected complex system. Furthermore, it is anticipated that a multi-omics approach will enable a better appreciation of the system’s resilience to urban physical and/or chemical changes and stress.

## 2. Materials and Methods

### 2.1. Water Sampling

**Figure 1:**
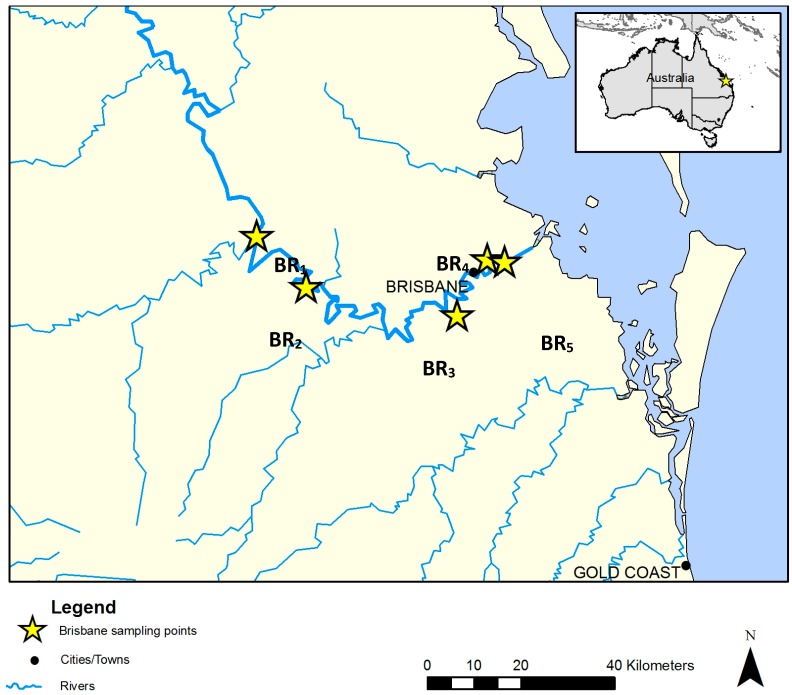
Map of the Brisbane River (BR) and the selected sampling sites (BR_1_–BR_5_).

Water samples were collected from five sites along the Brisbane River (BR), Queensland (Qld), Australia during the low outgoing tide in December 2013. For reference, the BR sampling sites are presented in Figure 1, are annotated with stars and designated BR_1_ to BR_5_. Triplicate samples were collected from each site at one sampling event, giving a total of 15 water samples. The in-situ measurements of temperature (°C), conductivity (mS/cm), pH, salinity (ppt), turbidity (NTU), and dissolved oxygen (mg·L^−1^) were made at the time of collection using a calibrated AQUAprobe AP-300 water quality probe (Aquaread, Broadstairs, Kent, UK). Moreover, each site was matched, by location and date, with sample sites from the Healthy Waterways “*Ecosystem Health Monitoring Program (EHMP)*” water quality monitoring program [29]. Of note, Healthy Waterways is a not-for-profit, non-government, membership-based organization working to protect and improve waterway health in South East Qld. Data from the EHMP matched sites were included in the analysis in order to expand the breadth of physico-chemical data collected.

At each site, triplicates of 10-liter water samples were collected from 30 cm below the water surface in sterile carboy containers. The water samples were then transported on ice to the laboratory, and processed within 6–8 h. Sample site characteristics and GPS coordinates are provided in Table 1. In addition, complementary data from the matched EHMP sites are provided in Table 2. Furthermore, site BR_1_ is located on the upper reaches of the BR. This site receives overflow of water from the Wivenhoe Reservoir. Site BR_2_ is located in a peri-urban non-sewered catchment. Site BR_3_ is at a major tributary of the Brisbane River. The catchment where site BR_3_ is located has residential and industrial developments and is serviced by a wastewater treatment plant (WWTP). Sites BR_5_ and BR_6_ are located on the lower reaches of the river, in highly urbanized areas and is tidally influenced. The catchment had not received any rainfall 7 days prior to sampling.

### 2.2. Water Quality Analysis

#### 2.2.1. Dissolved Organic Carbon

Samples were filtered through a 0.45 μm pore size (47 mm diameter) hydrophilic membrane (Durapore polyvinylidene difluoride, PVDF, Millipore, Tokyo, Japan), and the dissolved organic carbon in 30 mL of sample was determined in triplicate using a 820 TOC (total organic carbon) analyzer (Model: Sievers, GE Analytical Instruments Inc., Boulder, CO, USA).

#### 2.2.2. Trace Metals

A 10 mL aliquot of water from each site was filtered through a 0.45 µm pore size nitrocellulose membrane (Millipore) prior to acidifying to 2% with concentrated nitric acid (AR grade; Sigma Aldrich, Castle Hill, NSW, Australia). Each sample was prepared in triplicate, with three samples collected per site (*n* = 45, for all five EHMP sites). Aluminum (Al), cadmium (Cd), cobalt (Co), chromium (Cr), copper (Cu), iron (Fe), lead (Pb), nickel (Ni) and zinc (Zn) were determined using an Agilent 7700x quadrupole-type ICP-MS (Agilent Technologies, Mulgrave, VIC, Australia) equipped with an Agilent ASX-520 auto sampler. The instrument was operated in He-mode. The integration time was 0.3 s per mass, 1 point per mass, 3 replicates and 100 sweeps per replicate. All samples and standards were stored in Falcon tubes (15 mL) that were rinsed with 2% nitric acid, and kept at 4 °C until analyzed. All consumables were soaked in 10% nitric acid for at least 24 h and rinsed repeatedly with MilliQ and 2% nitric acid (in MilliQ water) before use.

#### 2.2.3. Enumeration of Fecal Indicator Bacteria (FIB)

The membrane filtration method was used for the isolation and enumeration of FIB. Serial dilutions of water samples were made in sterile MilliQ water, and filtered through 0.45-µm pore size nitrocellulose membrane (Merck Millipore, Bayswater, VIC, Australia). Dilutions were placed on modified membrane-thermotolerant *Escherichia coli* agar medium (modified mTEC agar, Difco, Detroit, MI, USA) and membrane-*Enterococcus* indoxyl-d-glucoside (mEI) agar (Difco) for the isolation of *E. coli* and *Enterococcus* spp., respectively. Modified mTEC agar plates were incubated at 35 °C for 2 h to recover stressed cells, followed by incubation at 44 °C for 22 h, while the mEI agar plates were incubated at 41 °C for 48 h [30,31].

### 2.3. Biomass Collection from Water Samples

Four sub-samples (2 L) of the 10 L parent sample were filtered through 0.45 µm pore size nitrocellulose membrane. Multiple membranes were used in case of membrane clogging due to sample particulates. The membrane(s) were immediately transferred into a sterile 15 mL Falcon tube containing phosphate buffered saline (Sigma-Aldrich, St. Louis, MO, USA). The sample tube was vortexed for 5 min to detach the microbial biomass from the membrane, followed by centrifugation at 4500 *g* for 15 min at 4 °C to obtain a pellet [32]. One sample tube was used for DNA extraction and subsequent metagenomics analysis. The remaining three tubes were used for metabolite extraction and subsequent community metabolomic analysis.

### 2.4. Metagenomic Analysis

#### 2.4.1. DNA Extraction

DNA was extracted from the pellet obtained from each water sample using the MO BIO PowerSoil^®^ DNA Isolation Kit (MO BIO Laboratories, Carlsbad, CA, USA) as per Ahmed et al. [33]. The extracted DNA samples were quantified using a ND-1000 spectrophotometer (NanoDrop Technology, Wilmington, DE, USA).

#### 2.4.2. PCR and Illumina MiSeq Sequencing

The V5 and V6 regions of the 16S rRNA gene were amplified using the primer set described previously [34]. Noting that the V5 and V6 region has been shown to provide equivalent diversity estimates to the V4 region, and is similar to full length 16S rRNA sequencing [35]. Amplicons from each sample were pooled in equal amounts. All samples were paired-end sequenced at a length of 300 nucleotides [nt] in each direction by the University of Minnesota Genomic Center (Minneapolis, MN, USA), using version 3 chemistry on the MiSeq platform. Raw data were deposited in the NCBI Sequence Read Archive under BioProject accession number SRP062949.

#### 2.4.3. Sequence Data Analysis

Sequence processing was performed using mothur software ver. 1.33.3 (http://www.mothur.org) [36]. Sequences were first trimmed to 150 nt and paired-end joined using fastq-join [37]. Quality trimming was performed to remove sequences with average quality scores <35 over a window of 50 nt, homo-polymers >8 nt, ambiguous bases, or mismatches to primer sequences. High-quality sequences were aligned against the SILVA database ver. 115 [38]. Sequences were further quality trimmed using a 2% pre-cluster [39,40], and chimera removal using UCHIME [41]. Assignment of OTUs was performed at 97% identity using the furthest-neighbor algorithm. Taxonomic assignments were made against the Ribosomal Database Project database ver. 9 [42]. For comparisons among BR sampling sites, sequence reads for each replicate were rarefied by random subsample to 25,000 (75,000 sequence reads per site).

### 2.5. Community Metabolomics Analysis

Overall, 45 samples were collected from five sample sites. Of note, each sample site was collected in triplicate and then subsequently analyzed in triplicate. All samples were derivatized prior to analyses by gas chromatography-mass spectrometry (GC-MS) as mentioned in previous studies [43,44,45]. Briefly, a 1.0 mL aliquot of ice cold methanol (LC grade, ScharLab, Sentemanat, Spain) and MilliQ water (50:50 *v/v*) were added to each sample pellet, then vortexed briefly before centrifugation at 572.5 *g* for 15 minutes at 4 °C. Adonitol (20.0 µg/mL, HPLC grade, Sigma-Aldrich, Castle Hill, NSW, Australia) was added as an internal standard. A 100.0 µL aliquot of the supernatant was then transferred to a fresh tube and dried in a centrifugal evaporator at 210 g and 37 °C (Model number: RVC 2-18; Martin Christ Gefriertrocknungsanlagen GmbH, Osterode, Germany).

#### 2.5.1. Sample Silyl Derivatization

For GC-MS analysis, dried samples were derivatized using 40.0 µL methoxyamine HCl (20 mg/mL in pyridine) followed by 70.0 µL *N*,*O*-bis(trimethylsilyl)trifluoroacetamide (BSTFA) in 1% trimethylchlorosilane (TMCS). Samples were then briefly vortexed before being transferred to GC-MS vials and microwaved for 3 min at 120 °C/600 W using a Multiwave 3000 system (Perkin Elmer Inc., Melbourne, VIC, Australia).

#### 2.5.2. Single Quadrupole GC-MS

Single quadrupole GC-MS was performed as previously reported [46,47]. Briefly, an Agilent 7890B gas chromatograph (GC) oven coupled to a 5977A mass spectrometer (MS) detector (Agilent Technologies) was used. The GC-MS system was fitted with a 30 m HP-5MS column, 0.25 mm internal diameter and 0.25 µm film thickness. Injections (1.0 µL) were performed in 1:10 split mode, with the oven held at an initial temperature of 70 °C for 2.0 min. The temperature was then ramped-up to 300 °C at 7.5 °C·min^−1^ and the final temperature (300 °C) was held for 5.0 min. The transfer line was held at 280 °C. Total ion chromatogram (TIC) mass spectra were acquired within a range of 45–550 *m/z*, with a 2.89 spectra·s^−1^ acquisition frequency. A solvent delay time of 7.5 min ensured that the source filament was not saturated or damaged. Data acquisition and spectral analysis were performed using the Qualitative Analysis software (Version B.07.00) of MassHunter workstation. Qualitative identification of the compounds was performed according to the Metabolomics Standard Initiative (MSI) chemical analysis workgroup [48] using standard GC-MS reference metabolite libraries (NIST 14, Fiehn and Golm) and with the use of Kovats retention indices based on a reference n-alkane standard (C8-C40 Alkanes Calibration Standard, Sigma-Aldrich, Castle Hill, NSW, Australia). For peak integration, a 5-point detection filtering (default settings) was set with a start threshold of 0.2 and stop threshold of 0.0 for 10 scans per sample.

### 2.6. Data Integration and Statistical Analysis

The metagenomics, metabolomics, trace metal and water quality data were subjected to further statistical analysis involving multivariate analyses of Principal Component Analysis (PCA) and Partial Least Square-Discriminant Analysis (PLS-DA) using SIMCA 14.1 (MKS Data Analytics Solutions, Uméa, Sweden). For the merging of these multiple datasets, the data was first imported, matched by sample location identifiers and log transformed. The analyses provided spatial distribution of various bacteria, their intracellular and extracellular metabolic activity and the relationships between the water quality parameters investigated.

## 3. Results

### 3.1. Physico-Chemical Data

Analysis of the physico-chemical parameters measured during sampling and the data available on the EHMP website revealed the level of degradation of the sampled system as a temporal snapshot. The salinity measurements of the sites indicate that BR_4_ and BR_5_ are above the recommended threshold for a freshwater ecosystem in the “*Australian and New Zealand Guidelines for Fresh and Marine Water Quality (AWA)*” [49] (threshold value of ca. 1.1 ppt). However, it is noteworthy to mention that these sites are influenced by the tide and the nearby estuary, although samples were collected during low tide. All the other sites were within the acceptable threshold for salinity (Table 1).

The AWA guidelines also state that lowland river systems have turbidity limits between 6–50 NTU. For the sites sampled, BR_3_ was observed to be above this threshold (137 NTU); the tidal influenced sites (BR_4_ and BR_5_) were also within this range but are considered high for estuaries/marine environments (which have a threshold of 0.5–10 NTU).

All sites were within the acceptable range stated in the guidelines with respect to pH (6.5–8.0). However, site BR_2_ was observed at the maximum range of the threshold (pH 8.0). For the EHMP derived data, chlorophyll a levels (5 μg·L^−1^) were observed to be within acceptable limits with the exception of site BR_1_ (6.3 μg·L^−1^). The total phosphorous (TP) (acceptable level ≤50 μg·L^−1^) was high for all the sites (above 50 μg·L^−1^), with sites BR_2_ (320 μg·L^−1^), BR_3_ (270 μg·L^−1^), and BR_4_ (140 μg·L^−1^) observed to contain exceptionally high TP when compared to the threshold value. Likewise for filterable reactive phosphate (FRP) (acceptable level ≤20 μg·L^−1^), all sites were observed above the guideline value, with sites BR_2_ (240 μg·L^−1^) and BR_3_ (260 μg·L^−1^) the highest. For total nitrogen (TN) (acceptable levels ≤500 μg·L^−1^), sites BR_2_ (880 μg·L^−1^) and BR_3_ (810 μg·L^−1^) were above the guideline value. Nitrogen oxide(s) (NO_x_) (acceptable level ≤60 μg·L^−1^) was high for all sites with the exception of BR_1_ (31 μg·L^−1^). Sites BR_2_ (500 μg·L^−1^), BR_3_ (550 μg L^−1^), BR_4_ (210 μg·L^−1^) and BR_5_ (100 μg·L^−1^) were observed to have significantly higher NO_x_ content than the threshold. Lastly, all sites were below the threshold regulatory limit for ammonium based nitrogen (NH_4_^+^) (<20 μg·L^−1^).

### 3.2. Trace Metals

Heavy metal pollution of waterways is typically associated with mining activities or discharges from manufacturing industries. Heavy metal pollution in water and sediments can have serious effects on the aquatic ecosystem and can make water unsuitable for livestock and/or human consumption. Furthermore, some animals (i.e., fish, shellfish and oysters) can also “bio-accumulate” metals [17], making them unsafe for consumption. As such, the concentration of metals in an urban stream is of interest, more so when the stream has the potential to further impact downstream fisheries in estuaries and marine environments.

In the context of this study, soluble metals in the sampled river were analyzed because they would most likely impact the planktonic biota (in terms of abundance and diversification) and their metabolism (i.e., metabolic output). However, metals bound within sediments and biofilms, although important, were considered outside the scope of this investigation and is the focus of future work.

All trace metals analyzed were below the trigger values set for freshwater aquatic ecosystems at the 90% level of protection of species in the guidelines. The trigger values were set at 80 μg·L^−1^ for aluminum, 0.4 μg·L^−1^ for cadmium, 1.8 μg·L^−1^ for copper, 5.6 μg·L^−1^ for lead, 13 μg·L^−1^ for nickel, and 15 μg·L^−1^ for zinc. The guidelines have no set limit for chromium, cobalt and iron [49]. However, as indicated in Table 3, site BR_3_ was observed to have elevated levels for all the metals analyzed. In particular, site BR_3_ was observed to have significantly higher concentrations of aluminum (2.01 μg·L^−1^) and iron (4.53 μg·L^−1^) compared to the up-stream and down-stream sampling sites. It also was observed to have slightly higher concentrations of cobalt (2.38 ng·L^−1^), chromium (1.0 ng·L^−1^), copper (8.1 ng·L^−1^), lead (7.6 ng·L^−1^) and nickel (36.9 ng·L^−1^). It is noteworthy to mention that site BR_3_ was located near a wastewater treatment plant and is located at the junction of a side stream that enters into the BR.

### 3.3. Fecal Indicator Bacteria (FIB)

Among the 15 water samples analyzed from the five sites, all samples yielded culturable *E. coli* and *Enterococcus* spp. The concentrations of *E. coli* and *Enterococcus* spp. in water samples ranged from 15–307 colony forming units (CFU) and 4–544 CFU per 100 mL of water, respectively (Table 4).

The concentrations of *E. coli* and *Enterococcus* spp. were much greater in water samples from sites BR_3_ and BR_4_ compared to the other sites. Furthermore, sites BR_3_, BR_4_ and BR_5_ were all classed as poor quality (Class D) using the Microbial Water Quality Assessment Category (MAC) framework outlined in the Australian recreational water guidelines. It should be noted that sites BR_1_, BR_2_, and BR_3_ all had evidence of wildlife within the sampling sites. Sites BR_4_ and BR_5_ are also known human recreational locations and may have wildlife present that was not observed at the time of sampling. These wildlife factors may influence the FIB measurements obtained [50,51]. Interestingly, in a parallel investigation by Ahmed et al. [33], it was observed that human fecal contamination was present at site BR_3_ using a microbial source tracking toolbox (MST) approach targeting human-wastewater-associated Bacteroides HF183 molecular markers [33]. In addition, Aves (avian) fecal contamination was observed using the avian-associated GFD molecular marker at sites BR_1_, BR_3_, BR_4_, and BR_5_; Bovinae (cow) fecal contamination was observed using the cattle-associated CowM3 molecular marker at site BR_2_ and *Equidae* (horse) fecal contamination was observed using a horse-associated molecular marker at site BR_4_.

### 3.4. Metagenomics

**Figure 2:**
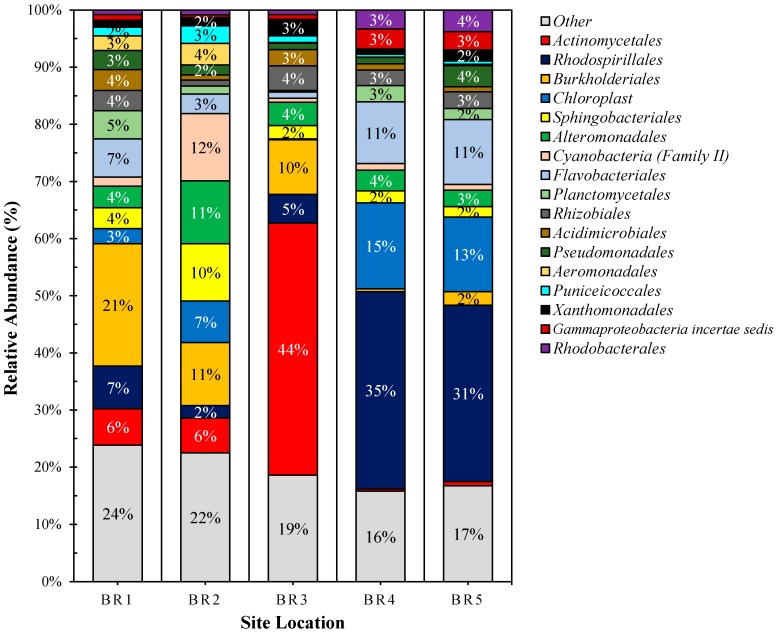
Bacterial order (top 17) profile of the BR sample sites. Note: ‘others’ represent orders less than 2% of the total sequence abundance.

**Figure 3:**
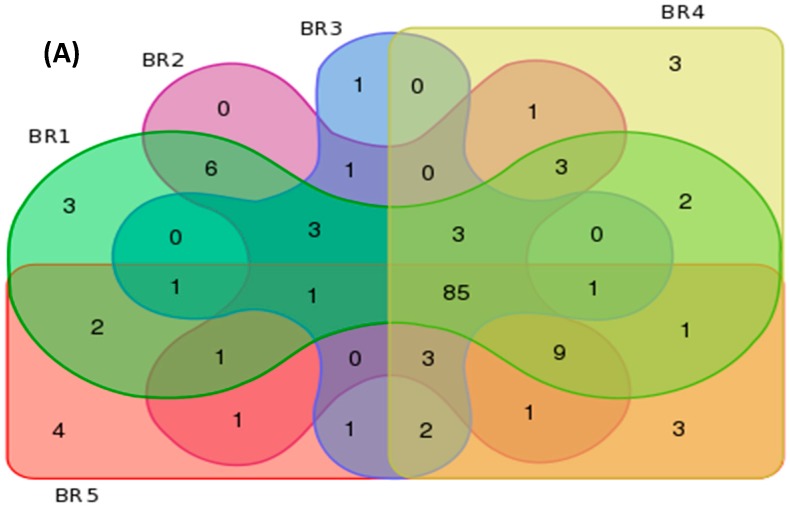

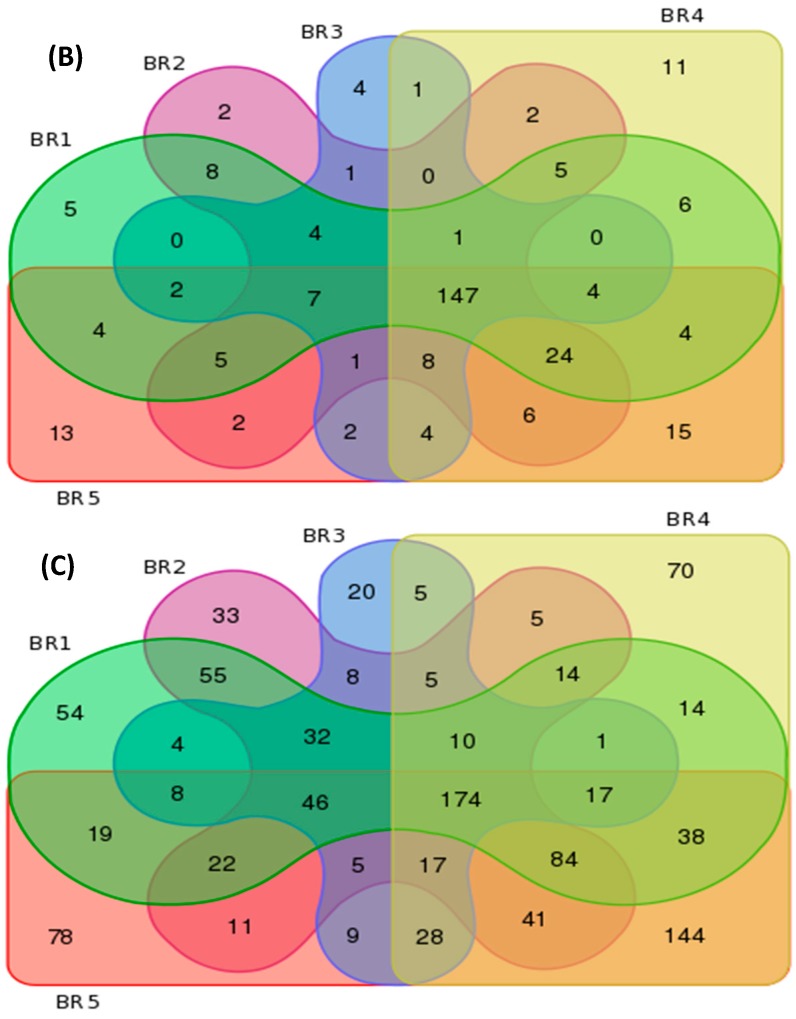
Bacterial metagenomics similarity and uniqueness characterization based on (**A**) order; (**B**) family and (**C**) genus.

The estimated Good’s coverage of the sample groups (75,000 sequence reads per site, with 3 independent samples subsampled to 25,000 sequence reads) ranged from 97% to 100%, with an average of 96% ± 1.2% among all samples. An average Shannon diversity index of 5.00 ± 0.24, OTU richness value of 1725 ± 172, and abundance-based coverage estimate of 4024 ± 1035 richness were observed among all samples. Table 5 provides a summary of the bacterial metagenomics data based on observed and unique features per order, family and genera for each sampled site. Figure 2 illustrates the bacterial order profile summary of the sampled sites and Figure 3 provides an overview of the site features in terms of similarity and uniqueness as presented as a Venn diagram.

The unique Family features for the sites were *Clostridiales incertae sedis*, *Desulfonatronaceae, Corynebacteriaceae*, *GpX*, and *Dermatophilaceae* for Site BR_1_; *Incertae Sedis*, *Aquificaceae* for site BR_2_; *Ktedonobacteraceae*, *Herpetosiphonaceae*, *Aerococcaceae*, and *Spirochaetales incertae sedis* for site BR_3_, *Brevinemataceae*, *Euzebyaceae*, *Dietziaceae*, *Thermoactinomycetaceae* 1, *Saccharospirillaceae*, *Cohaesibacteraceae*, *Dermacoccaceae*, *Clostridiaceae* 2, *Psychromonadaceae*, *Rubrobacteraceae*, and *Thiohalorhabdus* for site BR_4_; and, *Aquificales incertae sedis*, *Thermosporotrichaceae*, *Desulfarculaceae*, *Congregibacter*, *Cellulomonadaceae*, *Acholeplasmataceae*, *Bartonellaceae*, *Thermolithobacteraceae*, *Lactobacillaceae*, *Leuconostocaceae*, *Micromonosporaceae*, *Promicromonosporaceae*, and *Sphaerobacteraceae* for site BR_5_.

### 3.5. Community Metabolomics

The GC-MS analysis of the samples indicated a presence of 289 peaks per chromatogram, of which 54 were considered statistically significant (S/N ratio ≥50 with an adjusted *p*-value ≤ 0.05). Univariate and multivariate statistical tools such as *t*-test, Principal Component Analysis (PCA) and Partial Least Square-Discriminant Analysis (PLS-DA) were used to analyze the distribution and classification of the various metabolites. Due to the unsupervised nature of the data and the number of sample sites, PCA was observed as a less satisfactory method to discriminate between the metabolite distributions. As such, samples were processed further using Partial Least Square-Discriminant Analysis (PLS-DA). PLS-DA is used to examine large datasets and has the ability to measure linear/polynomial correlation between variable matrices by lowering the dimensions of the predictive model, allowing easy distribution between the samples and the metabolite features that cause the distribution.

**Figure 4:**
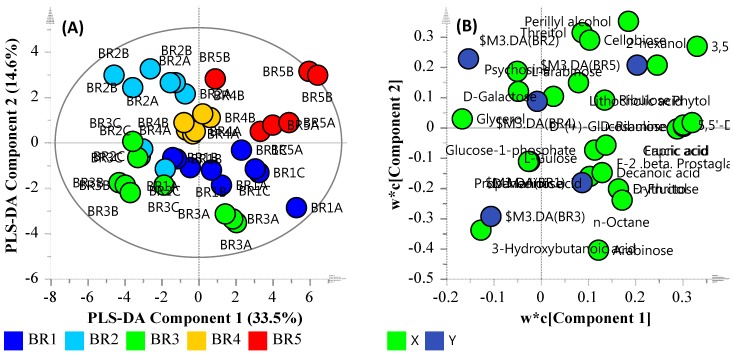
PLS-DA plot of the identified metabolites. (**A**) PLS-DA Score Scatter plot; (**B**) PLS-DA Loading Scatter plot.

**Figure 5:**
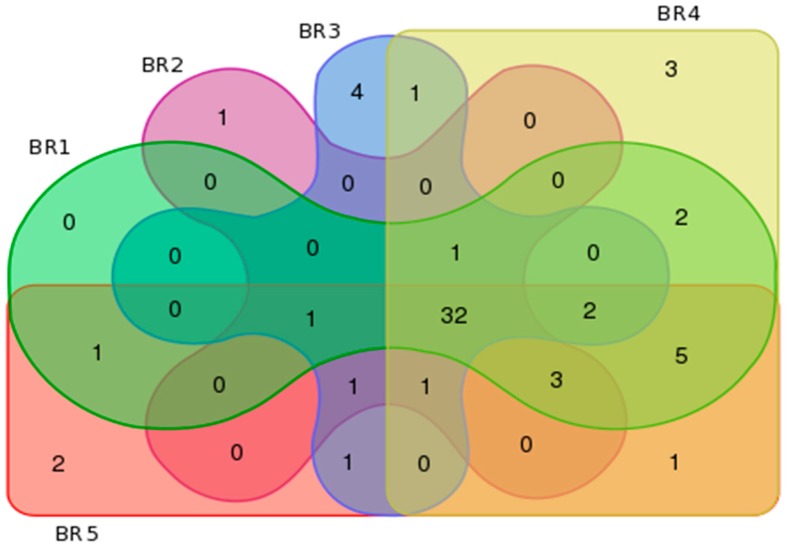
Metabolite similarity and uniqueness characterization.

The data quality of PLS-DA model was assessed by the linearity (*R*^2^*X*) and predictability (*Q*^2^), which were observed at 0.8294 and 0.565, respectively. These are indicative of a model that reasonably fits the data and has a weak/moderate predictive capability (~0.5). Figure 4A illustrates the PLS-DA score scatter plot of the metabolomic dataset groups (sample sites), and Figure 4B illustrates the loading scatter plot of the observed metabolites. The majority of the identified metabolites were sugars, fatty acids and amino acids. Secondary metabolites such as perillyl alcohol, lithocholic acid and phytol were also observed. As biological datasets tend to significantly vary from sample to sample, a distance of observation (DModX) analysis was also used to identify and eliminate any outliers. DModX is the normalised observational distance between variable set and X modal plane and is proportional to variable’s residual standard deviation (RSD). “DCrit (critical value of DModX)”, derived from the F-distribution, calculates the size of observational area under analysis. The DModX plot (not shown) data indicate that no samples exceeded the threshold for rejecting a sample. The threshold for a moderate outlier is considered when the sample DModX value is twice the DCrit at 0.05, which, in this instance, was 2.897 (DCrit  =  1.435). Table 6 lists the ‘identified’ significant metabolites after Benjamini-Hochberg adjustment. The unique metabolite features for the sites were Unknown Compound 13 (MW = 218.2) for site BR_2_; Xylitol (dTMS), l-Arabinose (4TMS), Unknown Compound 4 (MW = 325.2), and Unknown Compound 15 (MW = 189.1) for site BR_3_; Phytol mixture of isomers, Erythritol (4TMS) and d-Fructose (5TMS) for site BR_4_; and, Unknown Compound 18 (MW = 278.2) and Unknown Compound 9 (MW = 325.2) for site BR_5_. Figure 5 provides an overview of the site metabolite features in terms of similarity and uniqueness as presented as a Venn diagram.

### 3.6. Multi-Omics

As illustrated in the summary table (Table 7), an assessment of the water quality parameters in isolation is often difficult and tedious to decipher in terms of the system’s health and resilience; not to mention looking at the metagenomics and metabolomics data in isolation, due to the volume of data. An elevated result or a breach of the guidelines may not necessarily mean that the site or system is degraded. For example, the microbial indicators of the sites sampled suggest that sites BR_3_, BR_4_, and BR_5_ may pose a risk to human health (and were indeed classed as low quality). However, as illustrated in the study by Ahmed et al. [52] of the same samples, only site BR_3_ was observed to have a human wastewater signature. Likewise, sites BR_4_ and BR_5_ had elevated salinity levels according to the guidelines but it was noted that these sites were heavily influenced by the tide. As such, it is important to note that such data only provide a snapshot of the system at the time of sampling and may not represent the characteristics of the overall system at all times. One approach to overcome such problems is to sample the system more frequently (both temporarily and longitudinally). However, this will significantly increase the cost of analysis. An alternative approach that requires fewer samples to be collected is a multi-omics approach. Environmental multi-omics relies on a deeper analysis of the system being sampled in terms of bacterial diversity and metabolic output. Furthermore, it combines metadata to investigate relationships between sites. While it is ideal to do such an analysis over a period of time in order to establish seasonal trends, the study presented herein demonstrates its application and illustrates the added value of such an approach.

As such, in order to assess the entire system (from site BR_1_ through to BR_5_ from the perspective of heavy metals, physical and chemical parameters, metabolites and bacterial diversity), first the multiple datasets collected need to be collated and analyzed using a multi-omics approach in order to see if the data provide insight into the river system’s health. Investigating complex systems in isolation, whether it be analyzing measurements or sites in isolation, without consideration of upstream and downstream conditions, can result in an incorrect assessment of overall health or degradation. To this end, a series of PLS-DA plots were created in order to combine the multiple datasets presented herein. Each dataset was first matched by site name and log transformed in SIMCA to normalize the data. This enabled the data to be interrogated and provided a greater depth of analysis compared to investigating each site and parameter in isolation. The following section details such an assessment using the MAC characterization (i.e., Class A and D; which is also the same grouping as turbidity), the salinity data (i.e., high and low salinity) and MAC classification in combination with low salinity site data to categorize sites for comparison.

#### 3.6.1. Microbial Water Quality Assessment Category Class Assessment

**Figure 6:**
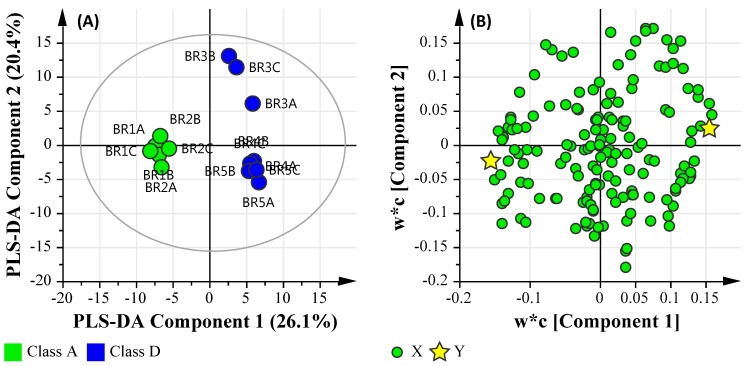
PLS-DA plot of the metadata and multi-omics datasets based the Microbial Water Quality Assessment Category class assessment. (**A**) PLS-DA Score Scatter plot; (**B**) PLS-DA Loading Scatter plot.

After the data were uploaded into SIMCA individually, matched by sample location identifiers and log transformed, they were then grouped based on the MAC category of ‘Class A’ and ‘Class D’. The resulting PLS-DA model was assessed by the linearity (*R*^2^*X* and *R^2^Y*) and predictability (*Q*^2^), which were observed at 0.584, 0.987 and 0.750, respectively. This is indicative of a model that reasonably fits the data and has a good predictive capability (>0.7). Figure 6A illustrates the PLS-DA score scatter plot of the combined datasets grouped based on MAC values (i.e., Class A and Class D), and Figure 6B illustrates the loading scatter plot of the observed parameters.

Using the MAC Class PLS-DA model, the dominant significant taxa classified at the class level for the ‘Class A’ pooled samples were *Acidobacteria*, *Alphaproteobacteria*, *Anaerolineae*, *Bacilli*, *Betaproteobacteria*, *Chlamydiae*, *Chloroflexi*, *Elusimicrobia*, *Fusobacteria*, *Gammaproteobacteria*, *Gemmatimonadetes*, *Holophagae*, *Ignavibacteria*, *Ktedonobacteria*, *Mollicutes*, *Negativicutes*, *Nitrospira*, *Opitutae*, *Spartobacteria*, *Spirochaetes*, *Thermodesulfobacteria*, *Verrucomicrobiae*, and *Zetaproteobacteria*. The dominant significant metabolic features were metabolites relating to carbohydrate metabolism (l-gulose, l-arabinose), glucagon signaling pathway (α-d-Glucose-1-phosphate, dipotassium salt dihydrate), and starch and sucrose metabolism (d-Cellobiose). Furthermore, no trace metals were correlated with the pool ‘Class A’ sample cohort.

In contrast, the dominant significant taxa classified at the class level for the ‘Class D’ pooled samples were *Armatimonadetes*, *Chlorobia*, *Chloroplast*, *Chrysiogenetes*, *Chthonomonadetes*, *Clostridia*, *Cyanobacteria*, *Deferribacteres*, *Dehalococcoidetes*, *Deinococci*, *Epsilonproteobacteria*, *Fibrobacteria*, *Flavobacteria*, *Lentisphaeria*, *Planctomycetacia*, *Sphingobacteria*, *Synergistia*, *Thermolithobacteria*, *Thermomicrobia,* and *Thermotogae*. The dominant significant metabolic features were metabolites relating to secondary bile acid biosynthesis (Lithocholic acid), carbohydrate metabolism (3,6-anhydro-d-galactose), fatty acid biosynthesis (capric acid), fructose and mannose metabolism (d-mannose), biosynthesis of unsaturated fatty acids (erucic acid methyl ester), and pentose and glucuronate interconversions (d-ribulose), in addition to chemical markers commonly found in human waste stream such as phytol mixture of isomers (manufacture of synthetic forms of vitamin E and vitamin K1), and osteoarthritis medication (d-glucosamine hydrochloride). Lastly, the trace metals of Al, Cr, Fe, Co, Ni, Cu, Zn and Pb were associated with the pooled ‘Class D’ sample cohort.

This suggests that ‘Class D’ pooled samples are correlated based on a number of factors, primarily bacteria that are known to cause or influence algae blooms (such as *Cyanobacteria*), organisms that lack aerobic respiration (*Clostridia*, *Synergistia*) and a number of green sulfur and non-sulfur bacteria (*Chlorobia, Thermomicrobia*), which are exacerbated due to the presence of pollutants (such as the presence of human waste stream indicators and heavy metals). Furthermore, bacteria capable of dehalogenating polychlorinated aliphatic alkanes and alkenes (*Dehalococcoidetes*) and organisms highly resistant to environmental hazards (*Deinococci*) were more abundant in Class D pooled samples. The presence of such organisms suggest the organisms within the sites are resistant to pollutants. However, the presence of *Fibrobacteria* suggests that commensal bacteria and opportunistic pathogens may also be present. In contrast, ‘Class A’ pooled samples were found to have organisms more commonly found in soil and aquatic environments, with no significant human waste-derived contaminants or metals present.

#### 3.6.2. Salinity Assessment

**Figure 7:**
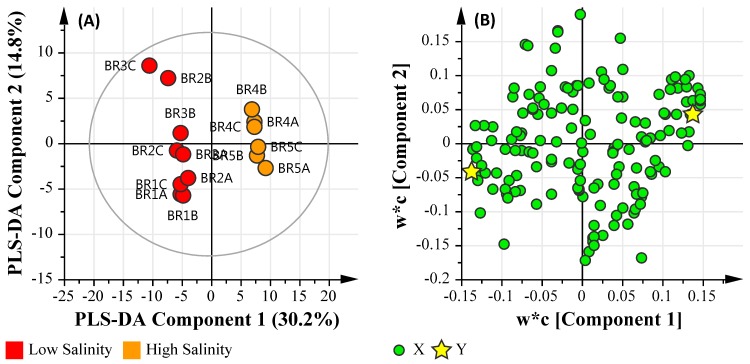
PLS-DA plot of the metadata and multi-omics datasets based the Microbial Water Quality Assessment Category class assessment. (**A**) PLS-DA Score Scatter plot; (**B**) PLS-DA Loading Scatter plot.

The data were grouped based on salinity data which was classed as ‘Low’ (<1.0 ppt) and ‘High’ (~30 ppt). The resulting PLS-DA model was assessed by the linearity (*R*^2^*X* and *R^2^Y*) and predictability (*Q*^2^), which were observed at 0.450, 0.983 and 0.911, respectively. This is indicative of a model that reasonably fits the data and has an excellent predictive capability (>0.9). Figure 7A illustrates the PLS-DA score scatter plot of the combined datasets grouped based on Salinity values (Low and High), and Figure 7B illustrates the loading scatter plot of the observed parameters.

Using the salinity class PLS-DA model, the dominant significant taxa classified at the class level for the ‘Low’ salinity sample sites were: *Acidobacteria*, *Alphaproteobacteria*, *Anaerolineae*, *Bacilli*, *Betaproteobacteria*, *Chlamydiae*, *Chloroflexi*, *Elusimicrobia*, *Fusobacteria*, *Gammaproteobacteria*, *Gemmatimonadetes*, *Holophagae*, *Ignavibacteria*, *Ktedonobacteria*, *Negativicutes*, *Nitrospira*, *Opitutae*, *Spartobacteria*, *Spirochaetes*, *Thermodesulfobacteria*, *Verrucomicrobiae*, and *Zetaproteobacteria*. The dominant significant metabolic features were metabolites relating to carbohydrate metabolism (l-gulose, butanoic acid), alanine metabolism (propanedioic acid), Biosynthesis of secondary metabolites (glycerol). Furthermore, Al, Cr, Zn and Pb were correlated with the ‘low’ salinity pooled cohort.

In contrast, the dominant significant taxa classified at the class level for the ‘High’ salinity sample sites were: *Armatimonadetes*, *Caldilineae*, *Chlorobia*, *Chloroplast*, *Chrysiogenetes*, *Chthonomonadetes*, *Clostridia*, *Cyanobacteria*, *Deferribacteres*, *Deinococci*, *Epsilonproteobacteria*, *Fibrobacteria*, *Flavobacteria*, *Planctomycetacia*, *Sphingobacteria*, *Synergistia*, *Thermolithobacteria*, *Thermomicrobia*, and *Thermotogae.* The dominant significant metabolic features were metabolites relating to secondary bile acid biosynthesis (lithocholic acid), carbohydrate metabolism (3,6-anhydro-d-galactose), fatty acid biosynthesis (capric acid), fructose and mannose metabolism (d-mannose), biosynthesis of unsaturated fatty acids (erucic acid methyl ester), osteoarthritis medication (d-glucosamine hydrochloride), and pentose and glucuronate interconversions (d-ribulose). In addition to chemical markers commonly found in human waste streams, such as Phytol, and perillyl alcohol (a monoterpene isolated from the essential oils of lavandin, peppermint, spearmint, cherries, celery seeds, and several other plants) were also detected. Lastly, Fe was associated with the pooled high salinity sample cohort. Like the previous assessment, the addition of salinity as a grouping highlights the presence of photosynthetic bacteria in addition to bacteria that are resilient to pollution sources.

#### 3.6.3. Microbial Water Quality Assessment Category Class A and Low Salinity Assessment

**Figure 8:**
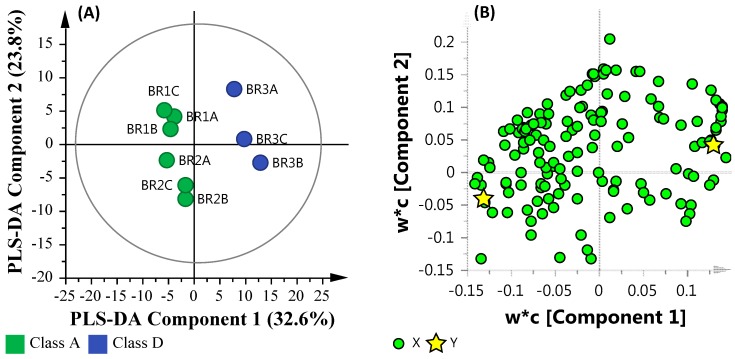
PLS-DA plot of the metadata and multi-omics datasets based the Microbial Water Quality Assessment Category class and low salinity assessment. (**A**) PLS-DA Score Scatter plot; (**B**) PLS-DA Loading Scatter plot.

As illustrated in Figure 6, sites BR_1_ and BR_2_ were grouped apart from BR_3_. In order to further analyze this sub-grouping, the ‘High’ salinity based sites (BR_4_ and BR_5_) were removed and a subsequent PLS-DA comparison was undertaken. Figure 8 illustrates the PLS-DA comparison based on Microbial Water Quality Assessment Category class and ‘Low’ salinity. The resulting PLS-DA model was assessed by the linearity (*R*^2^*X* and *R^2^Y*) and predictability (*Q*^2^), which were observed at 0.560, 0.964 and 0.657, respectively. This is indicative of a model that reasonably fits the data and has an average predictive capability (≥ 0.5).

This comparison highlights the increased presence of metals, short-chain fatty acids (SCFA) and sugars in site BR_3_ when compared with sites BR_1_ and BR_2_. Furthermore, the increased abundance of bacteria belonging to *Acidobacteria*, *Actinobacteria*, *Armatimonadetes*, *Chloroflexi*, *Chloroplast*, *Chrysiogenetes*, *Chthonomonadetes*, *Dehalococcoidetes*, *Fibrobacteria*, *Sphingobacteria*, and *Thermolithobacteria* suggests an environment that is capable of dehalogenating polychlorinated aliphatic alkanes and alkenes (*Dehalococcoidetes*) and the presence of *Fibrobacteria* suggests that commensal bacteria and opportunistic pathogens may also be present.

## 4. Discussion

Bacterial populations were observed to be affected by the nature of the sites sampled. BR_1_ site was observed to be rich in soil- and water-based bacteria, *Ralstonia* and *Bordotella* from *Burkholdericeae* family, mostly of wildlife/domestic animal sources and plant origin. Also, expectedly, a large number of *Actinomycetes* were also observed at BR_1_ and BR_2_ sites. The *Burkholderiaceae* population dropped at the agriculturally prominent site, BR_2_. Greater values for parameters such as temperature, pH, phosphates and nitrogen compounds resulted in a greater abundance of photosynthetic populations such as *Cyanobacteria* and other chloroplast-containing bacteria. The presence of cyanobacterial family II is possibly indicative of increased occurrence of sulphur compounds at BR_2_. It was observed that the populations of *Alteromonas* and related families within the *Alteromonadales* increased at BR_2_. This was unexpected as these bacteria are generally found in marine environments. However, it is probable that the tidal nature of the Brisbane River (86 km from the mouth) combined with high dissolved oxygen content [53] (9.3 mg·L^−1^) and added phosphorous salts (320 μg·L^−1^) might have resulted in higher *Alteromonas*, possibly due to the high phosphate metabolizing ability of organophosphorus acid anhydrolase (OPAA) (EC3.1.8.2) expression systems [54]. Due to the vicinity around wastewater treatment plants and other similar activities, site BR_3_ was expected to have greater abundances of *Actinomycetes*, *Streptomyces*, and *Frankia* and other related facultative anaerobic bacteria. The site was observed to have considerably greater turbidity with respect to previously reported values of about 50–60 NTU [53]. *Actinomycetes* are known to decay organic matter, especially in nutrient-rich environments such as wetlands [55] and various river samples [56], especially in association with *Sphingobacteria* around areas of human and animal activities. The co-occurrence of *Actinomycetes* with *Spingobacteria* was expected as it has been reported that the latter are efficient degraders of geosmin and 2-methylisoborneol, the compounds produced by *Actinomycetes*. These compounds have been reported to be responsible for increasing turbidity and odor of the riverine water system [57]. The current findings are therefore in line with previous reports, as greater turbidity was observed at site BR_3_ relative to sites BR_1_ and, especially, BR_2_. Similarly, greater turbidity at downstream sites of BR_4_ and BR_5_ may be partially attributed to less abundant sphingobacterial populations at those sites. It is also important to note that fecal coliforms, which comprises such organisms as *Escherichia coli*, *Klebsiella pneumoniae* and *Enterobacter aerogenes* (order *Enterobacteriales*) and faecal streptococci such as *Enterococcus* (order *Lactobacillales*) were not observed in significant numbers, although the study by Ahmed et al. [33] detected the presence of human and non-human fecal molecular markers.

The sites BR_4_ and BR_5_ are located very close to BR mouth, at the distances of about 22 km and 13 km, respectively. The area is considered as an inter-tidal zone, with a decreased flow-rate. The inter-tidal nature was associated with decreased levels of turbidity (15.3 NTU at BR_4_ with respect to 137.3 NTU at BR_3_) and dissolved oxygen. Due to the contribution of downstream oceanic and upstream sedimentation salts (from silts, soils, human and animal activities), inter-tidal zones, especially tidal flats have reportedly higher amounts of salinity. The higher content of deposited clay in this region also contributes towards increased salinity as compared to low clay soils or sands [58]. The high nutrient deposition combined with low flow rate and slightly anoxygenic nature of water at site BR_4_ and BR_5_ sites possibly resulted in increased populations of *Rhodobacteria* and *Rhodospiralles* such as *Rhodobacter*, *Acetobacter*, and *Azosirillium* spp. among others. Most of these bacteria are known to be chemo- and photo-autotrophic in nature and reportedly occur around Mangrove ecosystems (which are common around Brisbane). A significant decrease in nitrogen content at site BR_4_ and BR_5_ could be attributed to the nitrogen fixing and phosphate solubilisation abilities of these bacteria [59]. Other bacterial classes with a population increase were from Gamma-proteobacteria, with the order *Incertae sedis* members concentrated at site BR_4_ and *Pseudomonadales* (4%) at site BR_5_. It is very likely that the significant decrease in phosphates, nitrogen and turbidity was caused by the solubilization facilitated by *Pseudomonas* and related species at site BR_5_ [59].

It was noticed that the lower abundances of *Burkholderiales* at sites BR_2_ and BR_3_ were associated with decreased levels of sugars and sugar alcohols. Similarly, the greater abundances in *Cyanobacteria* at BR_2_ and *Actinomycetes* at BR_3_ are likely to influence the observed greater concentrations of SCFAs, such as butanoic and propanedioic acid. Such SCFAs enhance the biofilm formation ability of bacteria. In particular, exposure to lower concentrations of SCFAs, such as ca. 6 mM, enhances the biofilm formation ability of *Actinomycetes* bacteria [60]. Similarly, an increased level of erythritol could also be linked to bacterial biofilm formation. Although the contribution of erythritol to biofilm formation was observed to be less than SFCA, its greater concentration across all the sites may compensate for the deficit. Furthermore, it has been shown that erythritol, along with amino acids such as aspargine and phenylalanine, act as major contributory metabolites towards biofilm formation [61]. The abundance of SCFAs may also be indicative of mixotroph organisms able to utilize different SCFAs as organic carbon sources, either during growth or nutrient stress lipogenic phases [62]. Lastly, the sugar levels increased again at sites BR_4_ and BR_5_, very likely due to the increased fermentation caused by *Rhodobacteria*, *Rhodospirilli*, Gamma-proteobacteria and, especially, photosynthetic bacteria. As such, based on the metabolites observed, sites BR_2_ and BR_3_ have metabolites present that suggest a system that has potential to form biofilms and/or a community of mixotrophs that utilize SCFAs and that sites BR_4_ and BR_5_ are environments undergoing degradation.

## 5. Conclusions

Multiple characterizations of the BR system were performed by various genomic, ionic and metabolic methods. The metagenomics output indicated a presence of high levels of freshwater bacteria such as *Burkholdariales* and lower levels of *Actinomycetes* and *Rhodospirillae* in the upstream sites. In contrast, the population levels reversed in downstream sites, affected by salinity, pH and oxygen availability changes. Human interference was indicated by the increasing populations of *Actinomycetes* (BR_3_), including fecal bacteria and *Pseudomonadales* (BR_4_ and BR_5_). Greater abundances of these populations in downstream areas was also possibly caused by the increased levels of sugar alcohols, such as erythritol, SCFAs and aromatic amino acids, contributing heavily towards biofilm production. Overall, the multi-omics approach presented herein was able to provide a deeper insight into water quality contamination and riverine health in terms of metabolic and microbial properties that are not possible using traditional water quality methods. As such, a multi-omics based approach should be considered when characterizing complex environmental systems, in particular when assessing the impacts of agricultural practices, sewage treatment and environmental endpoints.

## Figures and Tables

**Table 1:** Sample site description and in-situ water quality characteristics. Water quality parameters were analyzed in triplicate (*n* = 3) and the relative standard deviation as a percentage (%RSD) are presented in the parentheses.

BR Site	EHMP Site	GPS Coordinates	Temp. (°C)	Conductivity (mS·cm^−1^)	Salinity (ppt)	pH	Turbidity (NTU)	DOC * (mg·L^−1^)	Site Characteristics	Suspected Source of Pollution
BR_1_	714	27°23′58” S; 152°36′26′′ E	21.7 (0.4)	0.39 (<0.1)	0.19 (<0.1)	7.14 (<0.1)	0.5 (1.7)	9.32 (0.8)	Rural	Wildlife, waterfowl, recreational activities.
BR_2_	711	27°29′51′′ S; 152°42′7′′ E	28.1 (<0.1)	0.53 (<0.1)	0.25 (<0.1)	8.00 (<0.1)	5.2 (0.1)	9.30 (0.4)	Rural	Cattle, horses, septic tanks, wildlife.
BR_3_	718	27°33′8′′ S; 152°59′31′′ E	26.6 (<0.1)	0.7 (<0.1)	0.32 (<0.1)	7.00 (<0.1)	137 (0.2)	8.88 (0.5)	Peri urban, tidally influenced	Wastewater treatment plants, waterfowls.
BR_4_	703	27°26′38′′ S; 153°3′3′′ E	25.7 (<0.1)	46.5 (<0.1)	30.17 (<0.1)	7.77 (<0.1)	15.3 (4.4)	4.35 (0.5)	Urban	Recreational activities, tidal influence.
BR_5_	702	27°26′56′′ S; 153°5′0′′ E	25.4 (<0.1)	46.9 (<0.13)	30.47 (0.3)	7.71 (<0.1)	11.6 (0.1)	5.02 (1.2)	Urban	Port, industrial activities, tidal influence.

**Note**: EHMP is defined as “*Ecosystem Health Monitoring Program*”. * DOC is defined as dissolved organic carbon.

**Table 2:** Sample site description and EHMP site matched water quality characteristics.

BR Site	EHMP Site	Chlorophyll a (μg·L^−1^)	Light Penetration (Secchi Depth, m)	Phosphorus as P		Nitrogen as N			
				FRP * (μg·L^−1^)	Total (μg·L^−1^)	Ammonia (μg·L^−1^)	Organic (μg·L^−1^)	Oxides (μg·L^−1^)	Total (μg·L^−1^)
BR_1_	714	6.30	0.45	31	61	<4	323	13	340
BR_2_	711	0.37	0.15	240	320	12	368	500	880
BR_3_	718	1.50	0.60	260	270	6	254	550	810
BR_4_	703	2.24	0.50	93	140	8	202	210	420
BR_5_	702	2.13	0.85	64	79	10	190	100	300

**Note:** EHMP is defined as “*Ecosystem Health Monitoring Program*”. * FRP is defined as filterable reactive phosphorus.

**Table 3:** Concentrations of metals in the water sourced from different sites and attributed to different sources. Values in the parenthesis denote standard deviations between the samples (*n* = 9).

BR Site	Concentration of Metals (RSD %)								
	Aluminum (Al, ng·L^−1^)	Cadmium (Cd, ng·L^−1^)	Cobalt (Co, ng·L^−1^)	Chromium (Cr, ng·L^−1^)	Copper (Cu, ng·L^−1^)	Iron (Fe, ng·L^−1^)	Lead (Pb, ng·L^−1^)	Nickel (Ni, ng·L^−1^)	Zinc (Zn, ng·L^−1^)
BR_1_	1.0 (>0.1)	<0.04	0.04 (4.7)	<0.02	3.0 (0.1)	<0.571	<0.06	<0.06	4.6 (2.3)
BR_2_	1.0 (>0.1)	<0.04	0.14 (5.3)	<0.02	3.1 (0.1)	32.0 (3.0)	<0.06	0.2 (0.1)	3.6 (0.6)
BR_3_	2008 (4.9)	<0.04	2.38 (2.4)	1.0 (0.1)	8.1 (0.1)	4529.7 (188.1)	7.6 (0.2)	2.8 (0.1)	36.9 (1.2)
BR_4_	1.0 (>0.1)	<0.04	0.15 (10.1)	<0.02	4.3 (0.6)	94.1 (59.3)	<0.06	0.4 (0.2)	6.2 (3.1)
BR_5_	11.0 (1.9)	<0.04	0.26 (3.4)	<0.02	4.0 (0.2)	234.7 (27.6)	<0.06	0.4 (0.1)	6.8 (1.5)

**Table 4:** Microbial water quality based on the various coliform counting methods. Values in the parenthesis indicate standard deviations (*n* = 9).

BR Site	Fecal Indicator Bacteria (FIB) ^a^		Recreational Microbial Water Quality Assessment ^b^			
	*E. coli*/100 mL Geometric Mean (Std. Dev.)	*Enterococcus* spp./100 mL Geometric Mean (Std. Dev.)	*Enterococcus* spp. (<33 CFU/100 mL (%))	*Enterococcus* spp. (>157 CFU/100 mL (%))	Standardized (95th Percentile ^c^)	Microbial Water Quality Assessment Category (MAC)
BR_1_	15 (4)	4 (2)	100	0	2	A
BR_2_	69 (18)	22 (5)	100	0	2	A
BR_3_	307 (49)	544 (85)	0	100	13,300	D
BR_4_	149 (25)	189 (24)	0	89	12,900	D
BR_5_	88 (16)	163 (37)	0	56	10,600	D

^a^ Determined using the geometric mean of nine samples (*n* = 9); ^b^ Recreational Microbial Water Quality Assessment calculated using *Enterococcus* spp. data following the NH&MRC “Guidelines for Managing Risks in Recreational Water”; ^c^ Calculated using the ranked method (*n* = 9).

**Table 5:** Summary of site bacterial metagenomics characterization.

BR Sites	Features per Group			Unique Features per Site		
	Order	Family	Genus	Order	Family	Genus
BR_1_	121	226	592	3	5	54
BR_2_	118	223	562	0	2	33
BR_3_	102	186	389	1	4	20
BR_4_	117	238	667	3	11	70
BR_5_	116	248	741	4	13	78

**Table 6:** Most significant metabolites from the sampled river sites identified by based on their fold change (FC), *p*-value and Adjusted (Adj.) *p*-values.

Compound	Description	Fold Change	*p*-Value	Adj. *p*-Value
l-Gulose (5TMS)	Monosaccharide sugar	2.885793773	3.60 × 10^−6^	5.00 × 10^−2^
l-Arabinose (4TMS)	Sugar	1.995251285	0.20237	3.82 × 10^−2^
Glycerol (3TMS)	Component of triglycerides and of phospholipids	1.850174576	0.41024	3.13 × 10^−2^
α-d-Glucose-1-phosphate, dipotassium salt dihydrate		1.792927932	0.85638	9.72 × 10^−3^
Psychosine sulfate (dTMS)	Lipid and intermediate in the biosynthesis of cerebrosides	1.744904803	0.049444	4.58 × 10^−2^
d-Cellobiose (1MEOX, 8TMS)	Disaccharide sugar	1.626482976	0.0061321	4.86 × 10^−2^
2-methyl-2-butenedioic acid (2TMS)	Carboxylic acid	1.535874614	0.4597	2.71 × 10^−2^
Prostaglandin F2β (1MEOX, 4TMS)	Arachidonic acid metabolites	1.196188199	0.1738	3.96 × 10^−2^
d-Threitol (4TMS)	End product of d-xylose metabolism	1.092677384	0.60615	1.74 × 10^−2^
d-Fructose (5TMS)	Monosaccharide sugar	1.041734383	0.4944	2.08 × 10^−2^
n-Octane	Component of Fatty acid metabolism	0.981496334	0.92237	3.47 × 10^−3^
Perillyl alcohol (dTMS)	Isolated from the essential oils	0.959935677	0.60615	1.67 × 10^−2^
Decanoic acid methyl ester (1TMS)	Constituent of many plants	0.956956828	0.44286	2.85 × 10^−2^
d-Arabinose (4TMS)	Intermediate in biosynthesis of lipopolysaccharide	0.893785022	0.41024	3.06 × 10^−2^
l-Tyrosine (3TMS)	Amino acid	0.834176724	0.87829	6.94 × 10^−3^
Erucic acid methyl ester (1TMS)	Fatty acids	0.790950415	0.11213	4.17 × 10^−2^
Capric acid (1TMS)	Fatty acids	0.790934596	0.11213	4.24 × 10^−2^
d-Glucose 6-phosphate (1MEOX, 6TMS)	Aminosaccharide	0.782548013	0.07339	4.44 × 10^−2^
Phytol (1TMS)	Constituent of chlorophyll	0.706423756	0.034756	4.65 × 10^−2^
Unknown Compound 10 (MW = 206.2)		0.662675401	0.2944	3.47 × 10^−2^
Erythritol (4TMS)	Sugar alcohol	0.659587922	0.24544	3.75 × 10^−2^
3,6-anhydro-d-Galactose (dTMS)		0.6094551	0.088429	4.38 × 10^−2^
d-Ribulose (1MEOX, 4TMS)	Monosaccharide sugar	0.317429352	0.014781	4.72 × 10^−2^
d-Mannose (dTMS)	Carbohydrate	0.27712129	0.58678	1.81 × 10^−2^
Lithocholic acid (2TMS)	Bile acid formed from chenodeoxycholate by bacterial action	0.203416434	0.58678	1.88 × 10^−2^
Butanoic acid methyl ester (1TMS)	Fatty acid methyl ester	0.153370303	0.96669	2.78 × 10^−3^
2-methylpropanedioic acid (2TMS)	Malonic acid derivative	0.152544336	0.34935	3.33 × 10^−2^

**Table 7:** Physico-chemical and microbial water quality summary.

BR Site	Monitoring Indicators													
	Physico-Chemical										Microbial			
	Salinity	Turbidity	pH	Cholorphyll a	TP	FRP	TN	NO_x_	NH_4_^+^	Metals	*E. coli*	*Enterococcus* spp.	FIB (combined)	MAC *
BR_1_	N	N	N	Y	Y	Y	N	N	N	N	L	L	N	A
BR_2_	N	N	N	N	Y	Y	Y	Y	N	N	L	L	N	A
BR_3_	N	Y	N	N	Y	Y	Y	Y	N	N	H	H	Y	D
BR_4_	Y	Y	N	N	Y	Y	N	Y	N	N	H	H	Y	D
BR_5_	Y	Y	N	N	Y	Y	N	Y	N	N	H	H	Y	D

Note: * MAC is defined as the Microbial Water Quality Assessment Category.
